# Untargeted Plasma Metabolite Profiling Reveals the Broad Systemic Consequences of Xanthine Oxidoreductase Inactivation in Mice

**DOI:** 10.1371/journal.pone.0037149

**Published:** 2012-06-18

**Authors:** Qiuying Chen, Hyeong-Cheon Park, Michael S. Goligorsky, Praveen Chander, Steven M. Fischer, Steven S. Gross

**Affiliations:** 1 Department of Pharmacology, Weill Cornell Medical College, New York, New York, United States of America; 2 Departments of Medicine, Pathology and Pharmacology, Renal Research Institute, New York Medical College, Valhalla, New York, United States of America; 3 Metabolomics Laboratory, Agilent Technologies, Santa Clara, California, United States of America; Auburn University, United States of America

## Abstract

A major challenge in systems biology is integration of molecular findings for individual enzyme activities into a cohesive high-level understanding of cellular metabolism and physiology/pathophysiology. However, meaningful prediction for how a perturbed enzyme activity will globally impact metabolism in a cell, tissue or intact organisms is precluded by multiple unknowns, including *in vivo* enzymatic rates, subcellular distribution and pathway interactions. To address this challenge, metabolomics offers the potential to simultaneously survey changes in thousands of structurally diverse metabolites within complex biological matrices. The present study assessed the capability of untargeted plasma metabolite profiling to discover systemic changes arising from inactivation of xanthine oxidoreductase (XOR), an enzyme that catalyzes the final steps in purine degradation. Using LC-MS coupled with a multivariate statistical data analysis platform, we confidently surveyed >3,700 plasma metabolites (50–1,000 Da) for differential expression in XOR wildtype vs. mice with inactivated XOR, arising from gene deletion or pharmacological inhibition. Results confirmed the predicted derangements in purine metabolism, but also revealed unanticipated perturbations in metabolism of pyrimidines, nicotinamides, tryptophan, phospholipids, Krebs and urea cycles, and revealed kidney dysfunction biomarkers. Histochemical studies confirmed and characterized kidney failure in *xor*-nullizygous mice. These findings provide new insight into XOR functions and demonstrate the power of untargeted metabolite profiling for systemic discovery of direct and indirect consequences of gene mutations and drug treatments.

## Introduction

Metabolomics, the systems biology of small molecules, has emerged in the post-genomic era as a powerful tool for profiling differences in the expression of structurally diverse molecules in complex biological mixtures [1]–[6]. In comparison to other “omics: technologies, metabolomics offers the most proximal approach for defining key differences between cell phenotypes. A significant challenge to metabolomics is the untargeted discovery of changes in metabolite expression that are hidden in large data matrices, acquired using high-throughput analytical procedures. Notwithstanding, untargeted profiling offers the potential to discover disease-associated and drug-induced changes in the expression of diverse small molecules in a biological fluid or tissue, revealing biomarkers that can be used to inform on disease progression or the efficacy of clinical treatments. In this regard, metabolomics can serve as an extension of traditional (targeted) clinical analyses, providing a far more comprehensive snapshot of the functional status of a complex biological system [7]. The resulting identification and quantification of disease and treatment-associated metabolites can trigger unanticipated hypotheses and novel mechanistic insights.

The ideal metabolomic platform would be able to accurately quantify the broad structural spectrum of small molecules that reside in cells, tissues and biofluids, at their biological levels – an elusive challenge. Notably, the extreme complexity of most biological sample matrices demands multiple metabolite separation modes for broad analytical detection and molecular identification to be effectively approached. To meet this challenge, recent developments in high-performance liquid chromatography (LC) separations have provided promising platforms for the rapid resolution of thousands of metabolites in biological extracts [8]–[13]. Combined with advances in MS instrumentation that enable rapid acquisition of high-resolution accurate mass spectra and chemometric platforms for automated data analysis and interpretation, effective metabolomic strategies for profiling and identifying diverse metabolites have emerged [14]–[17].

Xanthine oxidoreductase (XOR) is a molybdopterin-containing enzyme that catalyzes the two terminal steps in purine catabolism, oxidizing hypoxanthine initially to xanthine and finally to uric acid. XOR exists in two interconvertible forms that each yield ureate: xanthine oxidase (XO) and xanthine dehydrogenase. The distinction is that XO uses molecular oxygen as its electron donor and generates superoxide as a product, whereas xanthine dehydrogenase preferentially reduces NAD+ without superoxide generation. Xanthine dehydrogenase is considered to be the predominant physiological form of XOR in cells. Over the past two decades, there has been growing interest in the pathophysiological roles of XOR in a wide range of disease processes [18]–[24]. For example, upregulation of XOR activity has been implicated in various ischemic diseases and leads to hyperuricemia, among other manifestations of reperfused tissue injury [25]. Genetic deficiency of *xor* in mice has been linked to renal fibrosis and disease [26], [27].

In this investigation, we sought to inventory the metabolic consequences that occur in plasma metabolite levels in mice with a functional deficiency in XOR and evaluate the capability of an untargeted LC-MS-based platform for profiling both predicted and unforeseen changes. Although the direct enzymatic role of XOR in mammalian purine degradation is established from studies of the isolated enzyme [28]–[32], the inferred purine-independent actions of XOR [26]–[29], [33], [34] and secondary downstream metabolic perturbations that can arise from purine-dependent and -independent actions remain to be defined.

In this post-reductionist era of protein science, there is a growing appreciation for the idea that protein functions should be studied and understood in the context of native physiological microenvironments [35]. Since plasma provides the predominant reservoir of metabolites for cellular ingress and egress, and metabolites are the final downstream effectors of genes, the plasma metabolome can be used to globally inform on the organismal consequences of altered gene expression and drug treatments. Thus, global plasma metabolite profiling offers the potential to provide a broad overview of effects that may be induced by drug actions and altered gene expression, rendering plasma metabolite profiling as a promising technology for *in vivo* functional genomics/proteomics. We demonstrate in this report that untargeted plasma metabolite profiling, using LC-MS with advanced chemometric data mining, reveals predicted alterations in purine degradation, but also identifies unanticipated functions of XOR and discovers plasma biomarkers that inform on emergent kidney failure.

## Results

### Technical Assessment of Plasma Metabolite Profiling Performance

**Figure 1:**
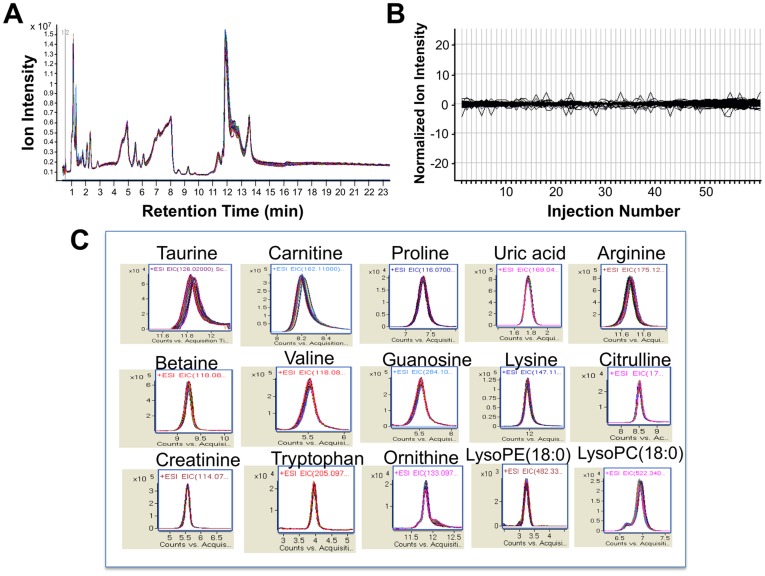
Technical reproducibility of plasma metabolite profiling. (A) Overlay of chromatograms acquired for 56 repeated analyses of a single plasma sample, assessing the consistency of ion count measurements. (B) Profile plot overlay of normalized extracted ion intensities for 374 distinct metabolites, quantified as a function of injection number for the 56 repeated plasma analyses depicted in *A.* Results depict flat run-to-run variation (mean CV = 6.51%) in the levels of the 374 metabolites with repeated analysis. (C). Peak overlay of 56 repeated assessments of extracted ion counts for detection of some typical plasma metabolites, demonstrating reproducibility of quantification.

Plasma, the reservoir of circulating metabolites, can provide a systems level read-out of the physiological state of an organism. To determine the technical reproducibility of untargeted analyses of plasma, we performed 56 repeat analyses on a 0.2 µl injected volume of a single human plasma (5 µl total injection volume, from a 25-fold diluted plasma sample) and analyzed the consistency of findings. Using data acquired by ANP chromatography with positive ion detection, an overlay of total ion chromatograms from the 56 repeat analyses revealed <10% run-to-run deviation (**Fig. 1A**). From these analyses, we tracked variations in the detection levels of 374 distinct metabolites that span the mass and chromatographic space of the dataset and additionally differ in ion abundance over 3-orders of magnitude (**Fig. 1B**). Considering the 56 repeated measurements of all 374 metabolites, normalized ion counts showed a mean coefficient of variation (CV) of 6.51%, median CV of 5.91% and high/low CV of 0.72% and 15.01%, respectively, considering all features. In accord with this relatively low overall CV for metabolite quantification, **Fig. 1C** shows extracted ion chromatography depicting the reproducibility in quantifying some typical plasma metabolites overlaying the 56 repeat measurements of cognate peaks. The high technical reproducibility of plasma metabolite quantification using this platform would predictably allow for confident identification of biologically-relevant changes that are ≥20% of control levels.

### Metabolic Pathway Perturbation Elicited by *xor* Gene Deletion

**Figure 2:**
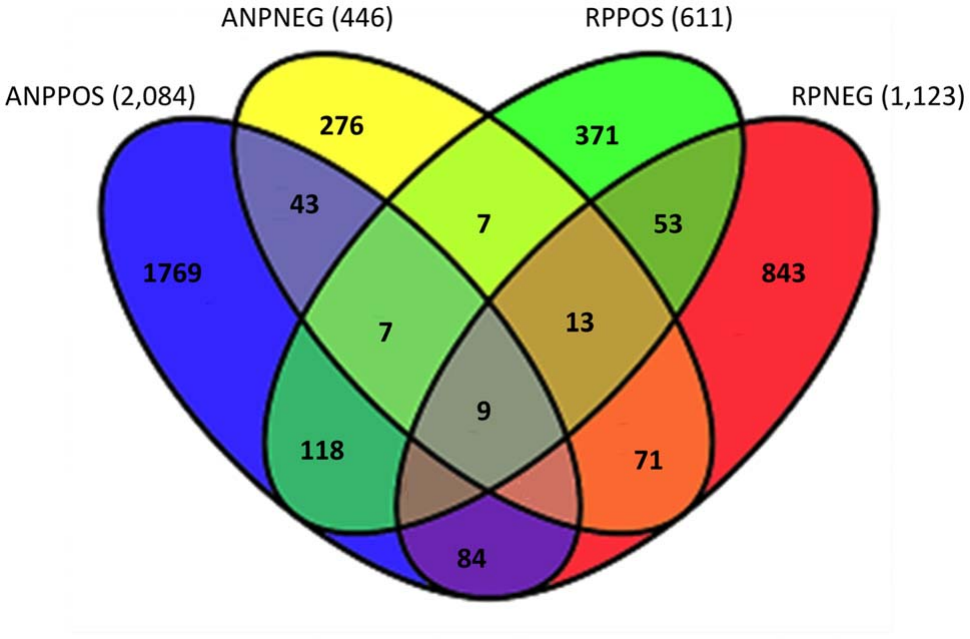
Broad murine plasma feature detection acquired by summed findings acquired using four different LC/MS detection modes. Chromatographic modes comprised aqueous normal phase (ANP) for hydrophilic molecules and reverse phase chromatography (RP) for hydrophobic molecules, with MS detection of both positive (POS) and negative (NEG) ions. As shown, all modes contributed substantially to feature coverage and only modest overlap was observed between detection modes. Notably, among the four detection modes, ANP-POS LC/MS allowed for quantification of the greatest number of features (2,004), followed by RP-NEG (1,123), RP/POS (611) and ANP-NEG (406). Combining data from all 4 acquisition modes, a total of ∼ 3,716 unique features were detected with 100% frequency in plasma from *xor* WT, KO, or both mouse groups.

Employing a multimode analytical platform for expanded feature coverage relative to that shown in **Fig. 1**, we analyzed plasma from *xor* WT and *xor* KO mice in both positive (POS) and negative (NEG) ionization MS detection modes, using both hydrophilic (ANP, aqueous normal phase) and hydrophobic (RP, reversed-phase) chromatographic separations. As shown in **Fig. 2**, each of the four data acquisition modes contributed substantially to feature coverage and only modest overlap was observed between detection modes. Among the four detection modes, ANP-POS LC/MS provided quantification of the greatest number of features (2,004), followed by RP-NEG (1,123), RP/POS (611) and ANP-NEG (406). Combining data from the 4 detection modes, a total of 3,716 unique features were quantified with 100% frequency in either *xor* WT, KO, or both. **Table 1** lists the verified identities of 52 metabolites found to be differentially-expressed in KO vs. WT plasma, with p<0.05 and fold-change >2.0. As indicated in **Table 1**, *xor* gene deletion was associated with a significant accumulation of some plasma features (denoted by a positive Log2 value) and a decrement in others (denoted by a negative Log2 value). In accord with expectations, untargeted plasma metabolite profiling revealed that *xor* gene deletion is associated with marked increases in circulating levels of xanthine, hypoxanthine, xanthosine and inosine, (XOR substrates and upstream metabolites in the purine salvage pathway), and depletion of uric acid and allantoin (the XOR product and downstream metabolite). Unexpectedly, *xor* gene deletion was also consistently associated with various other metabolic pathways, seemingly unrelated to purine metabolism; these include intermediates in metabolic pathways for pyrimidines, the tricarboxylic acid (Krebs) cycle, urea cycle, tryptophan, nicotinamide and phospholipids. Additionally, a series of small molecule biomarkers of kidney dysfunction [36] were observed to accumulate in plasma from *xor* KO mice, including creatinine, phenol, phenylsulfate, indoxyl, indoxyl sulfate, quinol, quinol sulfate, N-methyl-4-pyridone-5-carboxamide, N-Methyl-4-pyridone-2-carboxamide and hippuric acid. The observed array of predicted metabolite changes upstream and downstream of XOR enzyme activity affirmed the potential of metabolite profiling as a systems biology approach to identify gene functions, whereas unanticipated findings suggested the possibility of unappreciated XOR activities and unrecognized interactions of the perturbed purine salvage pathway with other metabolic pathways.

**Table 1:** Differentially-expressed plasma metabolites in *xor* KO vs. WT mice.

Metabolite/Pathway	MolecularFormula	Mass(measured)	Mass deviation (ppm)	RT(min)	Fold-change (Log2)*	p-value	Major detection mode
***Purine pathway***							
Xanthine	C5H4N4O2	152.0337	−2.7	1.621	16	6.1e−5	ANPPOS
Xanthosine	C10H12N4O6	284.0771	−4.8	1.759	16	6.1e−5	ANPPOS
Hypoxanthine	C5H4N4O	136.0389	−3.1	2.022	8.08	0.010	ANPPOS
Adenosine	C10H13N5O4	267.0971	0.0	2.476	3.09	0.021	ANPPOS
Inosine	C10H12N4O5	268.0813	−0.8	1.756	16	0.025	RPNEG
Uric acid	C5H4N4O3	168.0287	−2.3	1.678	−16	6.2e−4	ANPPOS
Allantoin	C4H6N4O3	158.0437	−0.1	1.574	−16	3.2e−4	ANPPOS
***Pyrimidine pathway***							
Uridine	C9H12N2O6	244.0694	−0.2	1.103	16	2.0e−5	RPNEG
Dihydrouridine	C9H14N2O6	246.0846	−2.6	1.695	2.27	0.0011	ANPPOS
Dihydrouracil	C4H6N2O2	114.0433	3.9	1.533	2.13	0.0042	ANPPOS
***Citric acid cycle***							
Malate	C4H6O5	134.0215	2.7	1.018	16	5.7e−4	RP-NEG
Aconitate	C6H6O6	174.0170	3.5	1.191	3.69	3.0e−4	RP-NEG
Fumarate	C4H4O4	116.0110	−0.5	1.050	3.72	0.022	RPNEG
Citrate	C6H8O7	192.0270	0.2	1.155	2.31	0.0050	RP-NEG
Ketoglutarate	C5H6O5	145.038	−3.5	1.057	5.64	8.2e−4	RP-NEG
Pyruvate	C3H4O3	88.0160	−1.2	1.144	1.94	0.0058	RP-NEG
***Tryptophan pathway***							
Kynurenic acid	C10H7NO3	189.0426	−1.3	1.695	3.82	0.0039	ANPPOS
Xanthurenic acid	C10H7NO4	205.0374	−0.1	3.288	3.35	0.042	ANPPOS
Indoxyl	C8H7NO	133.0529	−1.4	3.534	2.37	0.0086	RP-NEG
Indoxylsulfate	C8H7NO4S	213.0096	−1.7	3.536	2.19	0.0078	RPNEG
Tryptophan	C11H12N2O2	204.0892	2.2	4.420	−2.27	0.041	ANPPOS
***Urea cycle and related***							
Argininosuccinate	C10H18N4O6	290.1250	0.7	11.363	16	4.2e−5	ANPPOS
Fumarate	C4H4O4	116.0110	−1.5	0.755	3.72	0.022	RPNEG
Proline	C5H9NO2	115.0636	−3.7	6.996	1.29	3.4e−4	ANPPOS
Ornithine	C5H12N2O2	132.0899	−0.3	12.534	1.25	2.7e−4	ANPPOS
N-Acetylornithine	C7H14N2O3	174.0994	−3.1	8.599	1.59	0.045	ANPPOS
Citrulline	C6H13N3O3	175.0961	−2.3	8.601	1.22	0.031	ANPPOS
Homocitrulline	C7H15N3O3	189.1124	−3.9	8.576	1.72	5.7e−5	ANPPOS
Urea	CH4N2O	60.0329	−8.0	1.873	1.74	3.8e−5	ANPPOS
***Nicotinamide pathway***							
N-Methylnicotinamide	C7H8N2O	136.0635	0.0	11.295	2.24	0.033	ANPPOS
N-Methyl-2-pyridone-5-carboxamide	C7H8N2O2	152.0583	0.2	2.450	1.74	6.2e−5	ANPPOS
N-Methyl-4-pyridone-5-carboxamide	C7H8N2O2	152.0583	4.7	1.867	2.2	1.8e−4	ANPPOS
Niacinamide	C6H6N2O	122.0483	−2.7	1.846	−1.55	2.2e−5	ANPPOS
***Phospholipid metabolism***							
*Pantothenic Acid*	C9H17NO5	219.1111	−1.8	1.357	2.63	1.6e−4	ANPPOS
Myristoyl-lysophosphatidylcholine	C22H46NO7P	467.3011	0.5	7.696	2.21	2.0e−4	ANPPOS
Dioleoyl-phosphatidylethanolamine	C41H78NO8P	743.5447	2.4	1.748	2.14	1.0e−4	ANPPOS
Dioleoyl-phosphatidylcholine	C44H84NO8P	785.5937	−0.4	4.560	1.65	0.021	ANPPOS
Glycerolphosphocholine	C8H20NO6P	257.1035	2.8	12.172	−1.88	0.0080	ANPOS
***Renal disease markers***							
Creatinine	C4H7N3O	113.0590	2.5	6.776	16	1.0e−7	ANPPOS
Quinol	C6H6O2	110.0362	0.6	2.416	3.78	0.0039	RPNEG
Quinol sulfate	C6H6O5S	189.9929	1.0	2.417	2.49	0.0059	RPNEG
Hydroxyhydroquinone	C6H6O3	126.0314	2.7	1.670	1.08	0.0021	ANPPOS
Phenol	C6H6O	94.0420	−3.6	2.902	2.64	0.039	RPNEG
Phenylsulfate	C6H6O4S	173.9985	−1.4	2.901	2.29	0.046	RPNEG
Hippuric acid	C9N9NO3	179.0609	−1.9	1.131	2.55	0.0066	ANPNEG
2-hydroxyphenylacetate	C8H8O3	152.0472	0.1	7.986	1.21	0.048	RPNEG
***Other metabolites***							
Proline betaine	C7H13NO2	143.0948	−1.4	9.834	1.65	8.0e−4	ANPPOS
Myo-inositol	C6H12O6	180.0634	−3.8	3.345	1.84	4.8e−5	ANPPOS
3-Aminobenzoic acid	C7H7NO2	137.0481	−3.1	9.058	2.39	4.3e−4	ANPPOS
Hydroxyadipic acid†	C6H10O5	162.0530	−1.3	2.211	−1.36	0.0091	ANPPOS
1-Methylhistidine	C7H11N3O2	169.0854	−1.8	12.673	2.92	0.022	ANPPOS
S-Adenosylmethionine	C15H23N6O5S	399.1427	−3.5	13.01	−2.07	0.0019	ANPPOS

* Positive values indicate increased plasma levels in xor KO vs. WT, whereas negative values denote a decreased plasma levels in *xor* KO vs. WT.

† Identified as mixtures of 2–hydroxyadipic acid and 3-hydroxyadipic acid.

### Genotype-specific Metabolite Profiles Across Multi-treatment Groups

**Figure 3:**
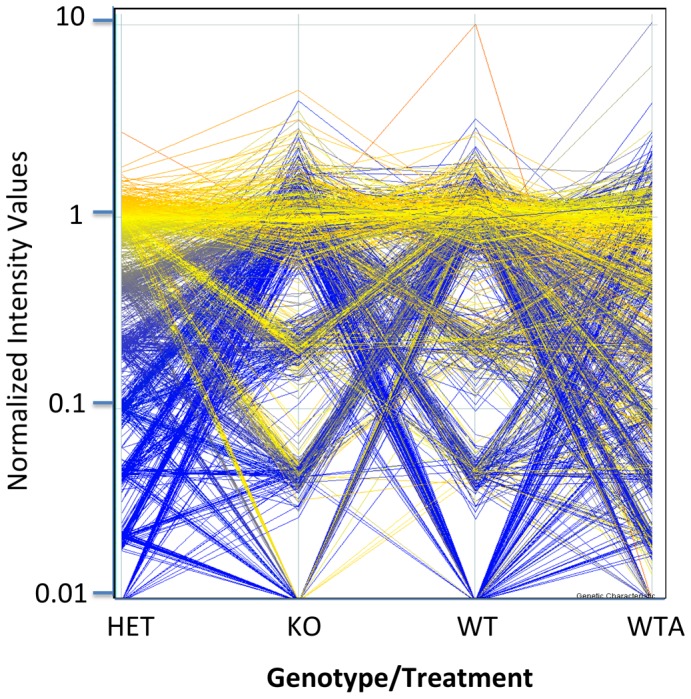
Patterns of change in murine plasma metabolite levels associated with XOR activity suppression. Striking similarities in plasma metabolite profiles are depicted in mice with deficient XOR activity (arising from either homozygous *xor* gene deletion or from allopurinol treatment; KO and WTA, respectively) vs. XOR-expressing mice (both *xor* HET and WT). Each line represents the levels of one of 1240 metabolites (quantified in all members of at least one group by ANP-POS), connected by its normalized abundance across groups. Color hues represent a heat map of normalized abundances (0.0–5.0), ranging from blue (cold) to red (hot).

Further studies were performed to assess the impact of *xor* gene dosage on the plasma metabolome. Toward this end, we compared plasma metabolite profiles from *xor* WT and KO mice with plasma from *xor* heterozygous mice (HET). Further, we sought to determine the extent to which the observed effects of *xor* gene-deletion on plasma metabolites would be recapitulated in *xor* WT mice after pharmacological inhibition of XOR activity, using the clinically-used drug, allopurinol (allopurinol treated group designated as *WTA*). Considering only ANP-POS metabolite profiling data in this expanded investigation, we observed a total of 8,360 unique aligned features in 18 plasma samples included in the study. Of the 8,360 observed features, 1,240 were quantified with 100% frequency in at least one group. **Fig. 3** depicts the changing patterns of expression of these 1,240 reproducibly identified plasma features across groups, comparing measurements from WT, HET, KO and WTA mice. These profiles are plotted in **Fig. 3** as the average abundance in each group. Results indicate striking similarities in the metabolite profile patterns of XOR-inactivated mice (whether allopurinol-treated or *xor*-null) and distinct differences from XOR-expressing mice (whether WT or HET). Among the 1,240 metabolites, relative levels of xanthine, hypoxanthine, uric acid and allantoin displayed pattern changes predicted for XOR protein expression patterns in *xor* WT, HET, KO and WTA. Numerous additional metabolites were observed to follow identical plasma expression patterns as that of xanthine, hypoxanthine, uric acid and allantoin, suggesting unappreciated metabolic linkages.

**Figure 4:**
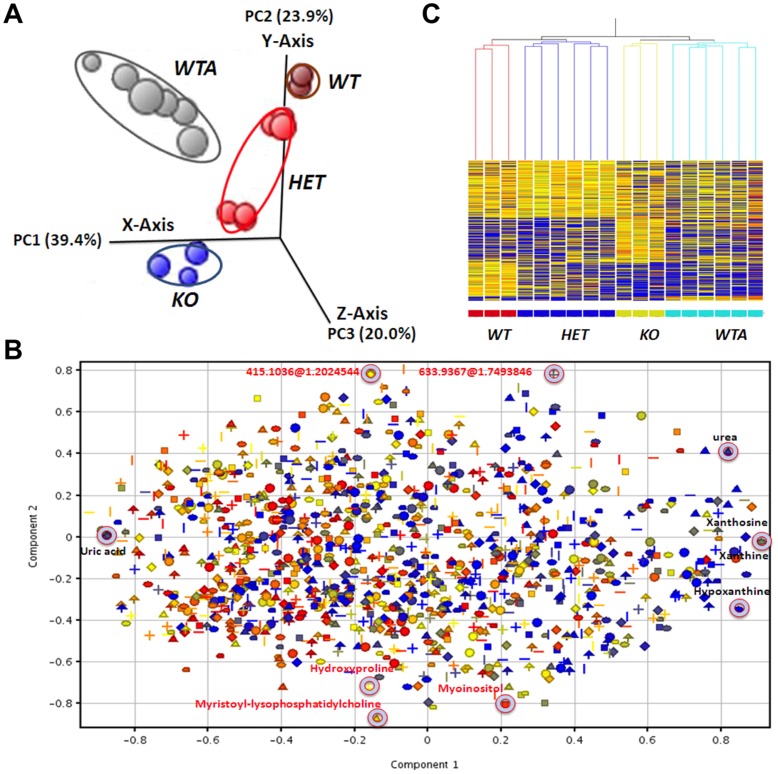
Separation of treatment groups by unsupervised principal component analysis (PCA) and hierarchical clustering analysis (HCA). (A) PCA score plot showing a three dimensional visualization of similarities and differences between each of the samples. Each data point corresponds to one sample with a 3-D (the first three principal components) projection of 1,240 metabolites. Clustering and separation of all samples indicates a gene dose trajectory in metabolite profile across groups. KO and WTA separated from WT along both PC1 (X-axis), which accounts for 39.4% of total variance. (B) PCA-loading plot showing 1,240 weighed metabolites contributing to the separation along PC1 and PC2 seen in score plot in *panel A*. Metabolites are colored by mass and shaped by compound. Identified metabolites contributing most strongly to the separation are indicated. Xanthine, hypoxanthine, xanthosine and urea were identified as up-regulated metabolites contributing dominantly to the separation along PC1, while uric acid and allantoin are among the down-regulated features that contribute most significantly to the separation along PC1. (C) Unsupervised HCA, based on the Pearson correlation, shows clustered within-group expression patterns for 1,240 quantified plasma features and significant differences in between-group expression. Feature intensity is depicted as a heat map, ranging from cold (blue) to hot (red) and was visualized using GeneSpring MS 1.2 software. Notably, mouse groups co-cluster with no-or-low XOR activity (*xor* KO and allopurinol-treated *xor* WT) and are clearly distinguished from mice with relatively higher XOR activity (*xor* HET and WT).

Because untargeted metabolomic studies are exploratory in nature and often result in large data sets, data analysis can benefit markedly from interpretation using multiple statistical and visualization tools. The goal of unsupervised pattern recognition is to identify and display natural groupings in the data without imposing any preconception about class membership. The PCA score plot, shown in **Fig. 4A**, provides a three-dimensional visualization of similarities and differences for all 1,240 plasma metabolites recognized in 100% of samples from at least one murine group. Each principal component (PC) represents the weighted linear combination of original LC/MS data, shown in the loadings plot (**Fig. 4B**), If two groups are found to differ in metabolite expression along the PC1 axis, then the loadings plot for PC1 can be used to determine which features contribute to the greatest extent in producing this difference. PCA findings show that *xor* WT and KO groups are clearly distinguished, reflecting distinct *xor* genotype-based differences in the respective plasma metabolomes. The *xor* HETs exhibited mixed pattern changes in their metabolite profiles, with 3/6 more closely resembling WT and the remaining 3/6 resembling KO. In contrast, *xor* WTA and KO are essentially inseparable along PC1, but clearly distinguished along PC2, reflecting a predominant component of XOR phenotype (activity) similarity in this parameter, despite genotype dissimilarity. This suggests that the genetic background of WTA may be inferred from PC2, while the influence of XOR inactivity may be revealed by PC1. Indeed, the dual contribution of XOR activity (PC1) and *xor* genotype (PC2) may be explicitly projected and visualized by the PCA score plot. It can also be seen in **Fig. 4A** that there is a clear trajectory from WT to HET, then KO, consistent with progressive changes in metabolism with increasing degrees of *xor* gene inactivation. The extent to which individual metabolites contribute to the changes along PC1 and PC2 can be evaluated by consideration of the loadings plot, depicted in **Fig. 4B**. Importantly, uric acid, xanthine, xanthosine, hypoxanthine, methylnicotinamide were found to be among the top loading metabolites for PC1, while glycerophosphotidylcholine, ceramides, myoinositol and several metabolites with undefined molecular structures were found to have the largest impact on PC2 (i.e., contribute most profoundly to observed metabolic differences in *xor* KO vs. WT mouse plasma).

Consistent with unsupervised pattern recognition found by PCA, the HCA dendrogram also showed reproducible patterns of within-group similarity and between-group differences in the 1,240 plasma features that could be quantified in all members of at least one group (**Fig. 4C**). Notably, groups sharing the phenotype of XOR inactivity (i.e. *xor* KO and WTA mice) exhibited significant and substantial correlation in their patterns of metabolite expression, clearly distinguishing these groups from mice that express active XOR (i.e. *xor* HET and WT mice).

**Figure 5:**
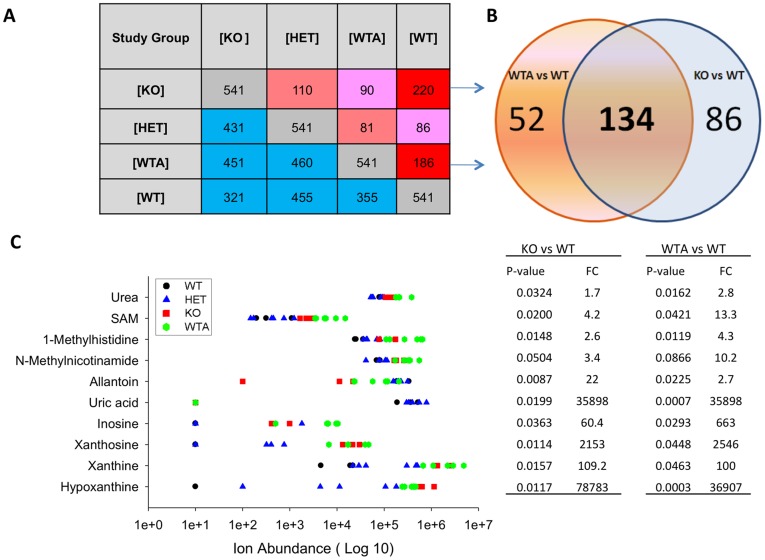
Statistically significant XOR activity-dependent differences in mouse plasma metabolite expression across groups. (A) Welch one-way ANOVA with Turkey Posthoc testing shows 541 statistically different metabolites in a group-by-group comparison (corrected, P<0.05). Numbers in pink and red cells denote significant and very significant metabolites between two groups, respectively. Blue denotes the number of metabolites showing no difference between two groups, and grey refers to the total number of differentially-expressed metabolites across all groups. (B) Venn diagram of the 220 and 188 differentially-expressed metabolites in KO and WTA compared to WT. Notably, KO and WTA share 134 differentially-expressed metabolites in common, compared to WT. (C) Differentially expressed metabolites showing similar changes in KO and WTA compared WT. Data points refer to metabolite ion abundance from 4 groups: WT (black circles), HET (blue triangles), KO (red squares) and WTA (green diamonds). The abundance of undetectable level of metabolites were assigned to 10 and plotted as one overlaid symbol. Identical metabolite abundance was plotted as one overlaid symbol. P-values comparing KO vs. WT and WTA vs. WT were calculated based on a 2-tailed Student’s t-test. Fold-changes were calculated based on the ratio of means for each group.

Welch ANOVA with Tukey’s HSD (Honestly Significant Difference) for *post hoc* testing revealed a total of 541 statistically different features in a group-by-group comparison of KO, WTA, WT and HET mice (**Fig**. **5A**). KO and WTA showed the greatest number of feature differences from WT. Of 220 and 186 statistically-significant feature differences in KO vs. WT and WTA vs. WT, respectively, 134 were changed in common (**Fig.** **4B**). These 134 conserved plasma metabolite feature changes that arise in association with suppressed XOR activity – irrespective of whether XOR is suppressed by gene deletion or pharmacological inhibition - suggests that these metabolic perturbations specifically arise from XOR enzyme inactivity. **Fig. 5C** shows the identity of metabolites found to be consistently upregulated or downredulated in both KO and WTA mouse plasmas, relative to WT. Apart from the anticipated changes in purine metabolites (i.e. xanthine, hypoxanthine and uric acid), we observed metabolites that are methylated or involved in methyl group transfer to be consistently upregulated in KO and WTA mouse plasma (i.e., S-adenosylmethionine, N-methylnicotinamide, and 1-methylhistidine). Additionally, KO and WTA mice exhibited a series of differentially expressed metabolites showing opposite changes, compared to WT, suggesting that these differences do not arise as a simple consequence of XOR enzyme inactivity (**Fig. S1**). These discordant features included glycerophosphocholines, ceramides and some of the urea cycle and polyamine pathway metabolites, which were significantly elevated in plasma from *xor* KO mouse plasma, but unchanged after allopurinol treatment (relative to WT).

### Physiological Correlations

**Figure 6:**
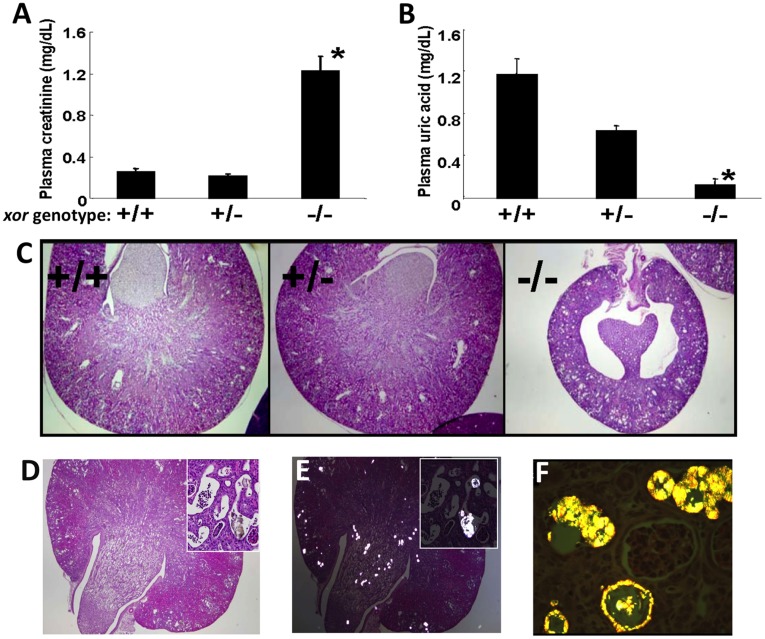
Comparison of renal function and histology in *xor*−/−, *xor*+/− *xor*+/+ mice. Renal function was assessed based on day 21 levels of plasma creatinine (A) and uric acid (B). Bars denote mean values +/− SEM; n = 3–4 mice. Asterisks denote p<0.05, compared to *xor* wild type. (D) H&E stained full thickness kidney section of a typical kidney from a *xor* KO mouse. Pathologic findings varied from focal deposits in mildly dilated cortical and papillary tubules to markedly attenuated renal parenchyma with large patchy scars, the latter most prominent in the areas with crystalloid deposits shown in the panel inset (10x original magnification). Note intratubular neutrophils frequently surrounding the crystalloid deposits in the tubular lumina mixed with necrotic debris (E) Panel D, viewed under polarized light, revealing the distribution of intratubular crystalloid deposits. (F) H&E stained kidney sections, visualized under polarized light with a yellow filter, 100 × original magnification. Brightly birefringent intratubular crystoliths were frequently observed, penetrating the tubular epithelial cells with apparent damage.

Untargeted plasma metabolite profiling revealed biomarkers consistent with kidney failure. To investigate the veracity of this possibility, we compared renal function and histology in *xor* KO mice vs. *xor* HET and WT mice. Using standard assays for renal function, *xor* KO mice exhibited conspicuous kidney failure at 14 days of life, when plasma was acquired for metabolite profiling. Kidney failure was evidenced by a marked elevation of plasma creatinine (1.22±0.15 mg/dL vs. 0.25±0.03 mg/dL in WT; (**Fig. 6A**) concomitant with severe hypouricemia (**Fig. 6B**). Additionally, kidney size was markedly diminished at 14 days, compared to *xor* WT and HET littermates (**Fig. 6C**). The build-up in plasma xanthine and hypoxanthine that we observed by untargeted metabolite profiling in *xor* KO mice was associated with the deposition of birefringent crystalloid concretions in the renal tubules, focally present as sludge in the renal pelvis (**Fig. 6D–F**). These crystalloids were occasionally observed to penetrate tubular epithelial cells, but were mostly intraluminal and partially-to-completely obliterative (i.e., resulting in variable degree of tubular dilation, predominantly affecting the outer cortex or the papillary tips). There was also a variable degree of papillary attenuation, suggestive of obstructive uropathy. In contrast, wildtype mice showed no significant kidney pathology and heterozygote mice exhibited mild focal simplification of glomerular capillary tufts with much more modest cortical tubulointerstitial scarring. Taken together, these analyses verified aberrant kidney structure and function in *xor* gene-deleted mice, in accord with predictions based on observed metabolic biomarkers of kidney dysfunction inferred from untargeted plasma profiling analysis.

## Discussion

XOR is not only the terminal enzyme in purine metabolism, catalyzing the oxidation of purine metabolites to uric acid, but it has also been implicated as a determinant of adipogenesis and peroxisome proliferator-activated receptor-γ activity [33], cyclooxygenase-2 gene expression [26], catalytic conversion of nitrate and nitrite to nitric oxide [29], [34], catalytic hydroxylation of a wide range of N-heterocyclic and aldehyde substrates [29], and may also provide an endogenous source of ROS formation for cell signaling [37], [38]. XOR activity is subject to both pre-transcriptional and post-transcriptional control by mechanisms that are modulated by hormones, cytokines and oxygen tension [29]. Given the multifunctional and poorly-defined systemic roles of XOR, we employed global plasma metabolite profiling in attempt to define XOR functions in mice and additionally, to assess the efficacy of untargeted profiling as an effective discovery approach. Taken together, our findings revealed unexpectedly broad XOR activity-dependent alterations in the plasma metabolome, uncovering primary, secondary, and tertiary biochemical derangements that apparently arise from deficient XOR enzyme activity. Further, plasma metabolome derrangements in *xor*-deleted mice indicated end-stage kidney failure, which was confirmed by histochemically-defined renal pathologies and marked hypercreatinemia and hypouricemia. Notably, *xor* gene KO was associated with interstitial fibrosis in the kidney and renal tubular accretions containing xanthine/hypoxanthine crystalloid matter (**Fig. 6**), consistent with a prior report [27].

**Figure 7:**
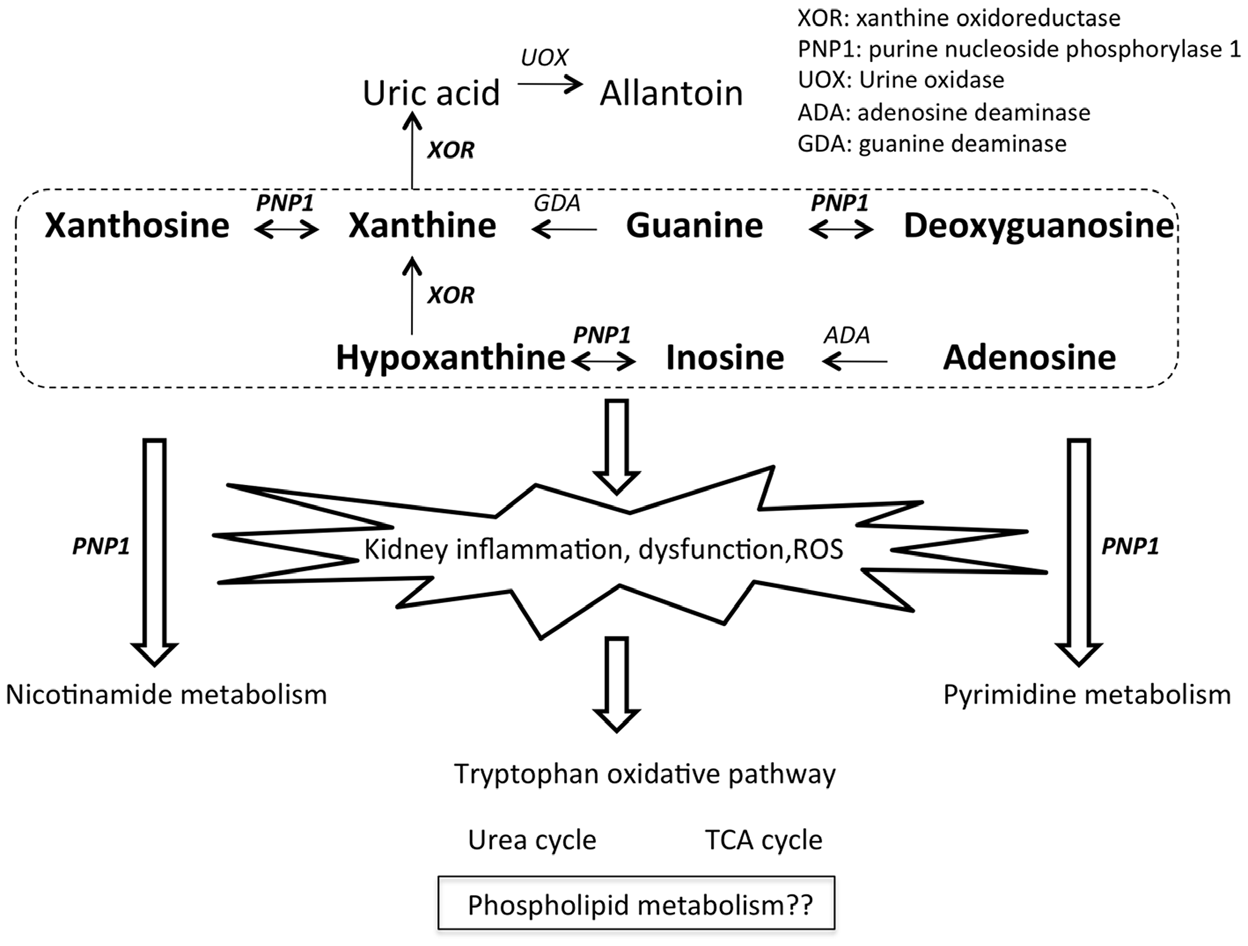
Schematic model for XOR insufficiency-induced primary, secondary and tertiary metabolic consequences, culminating in kidney failure. The present study found metabolites in bold to be upregulated in *xor* KO mice and non-bold metabolites downregulated. This model provides a framework that seeks to reconcile observed systemic consequences of *xor* gene KO on the murine plasma metabolome – see *Discussion* for details.

A schematic model is presented in **Fig. 7** that seeks to link primary, secondary and tertiary metabolic events that arise from *xor* gene deletion, as molecular underpinnings for renal pathophysiological consequences. In addition, a curated metabolic metabolic network that quantifies XOR gene-deletion induced changes is shown in supplemental **Fig. S2**. Primary metabolites that are upstream and downstream to XOR in the purine salvage pathway (i.e., xanthine, hypoxanthine and uric acid) were consistently identified in our study by untargeted profiling as among the most influential features that distinguish the plasma metabolome of both *xor* KO and allopurinol-treated mice from WT mice. We posit that the accumulation of primary XOR substrates (i.e., xanthine and hypoxanthine), drives an accelerated flux through the purine salvage pathway, accounting for substantial observed increases in secondary purine metabolite levels in plasma (i.e., xanthosine, inosine, adenosine and derived species). Accumulated xanthine and hypoxanthine provide abundant substrates for purine nucleoside phosphorylase 1 (PNP1), a purine salvage pathway enzyme that catalyzes the reversible phosphorolysis of purine nucleosides and inorganic phosphate, yielding the corresponding purine bases and ribose-1-phosphate. Notably, in addition to its service in the purine salvage pathway, PNP1 also catalyzes the inter-conversion of deoxyuridine to uracil (pyrimidine pathway) and N-ribosyl-nicotinamide to nicotinamide (nicotinamide metabolism). Given this involvement of PNP1 in multiple purine-independent pathways, elevated levels of secondary purine metabolites would predictably compete for the occupancy of PNP1, thereby interfering with the metabolism of other biomolecules that similarly rely on PNP1– this includes aberrant levels of pyrimidine and nicotinamide intermediates, considered in **Fig. 7** to be a tertiary metabolic consequence of XOR insufficiency. Notably, as shown in **Table 1**, pyrimidine and nicotinamide pathway metabolites were significantly perturbed in *xor* KO mice and this finding extended to allopurinol-treated *xor* WT mice. Competition for PNP1 by accumulated purine intermediates may also explain a prior report demonstrating significantly altered levels of uridine, uracil, cytosine, and nicotinamide in *xor*-deleted *Drosophila* [39].

The molecular basis for renal failure in mice with suppressed XOR activity is obscure, but is presumed to be triggered by primary, secondary, or tertiary metabolic perturbations that result from XOR deficiency, perhaps further enhanced in *xor* KO mice by a defect in kidney development. Notably, beyond the clinical gold standard biomarkers of kidney failure, creatinine and urea, untargeted plasma metabolite profiling identified numerous additional upregulated levels of biomarkers that are associated with kidney failure – these include phenols, indoxyl and its phase-II metabolite indoxylsulfate, quinol and its phase-II metabolites quinol sulfate [40] and quinol glunuronide, hippuric acid, hydroxyphenylacetate, trimethylamine-N-oxide. Polyamines, found at elevated level in renal disease patients’ plasma [41], were also significantly elevated in *xor* KO mice. Indoxyl sulfate is a protein-bound uremic toxin that is mainly produced by gut bacteria-mediated decomposition of dietary tryptophan in the intestine and accumulates in the plasma of patients with chronic kidney disease [40]. Serum indoxyl sulfate was shown to be associated with mortality in chronic kidney disease patients [42]. Impaired clearance of uremic toxins by the kidney results in their accumulation in plasma, with the potential to trigger systemic oxidative stress. Accumulation of these uremic toxins in plasma of *xor* KO mice may be either a cause or effect of renal kidney failure.

Less well explained metabolite changes associated with XOR inactivation in our study included increases in the apparent activity of the oxidative tryptophan/kynurenine pathway, TCA cycle and urea cycle (**Table 1**). Prior literature suggests that these metabolic perturbations could arise from kidney failure, possibly triggered by primary, secondary or tertiary metabolic consequences of insufficient XOR activity. For example, it has been shown that kynurenic acid, xanthurenic acid and kynurenine 3-hydroxylase activity are significantly increased in chronic renal failure in patients [43]–[45]. Moreover, the accumulation of L-kynurenine and its degradation product was found to be proportional to the severity of renal failure and correlated with the plasma concentration of renal insufficiency biomarker, creatinine [43]. Similarly, plasma levels of urea and the urea cycle intermediates ornithine and citrulline are elevated in renal failure patients, while arginine and aspartate have been shown to remain relatively constant [46]. We found that some urea cycle intermediates are also elevated in *xor* KO mouse plasma (ornithine, citrulline and argininosuccinate), along with some downstream metabolites (polyamines and proline), while levels of arginine and aspartate did not change (**Table 1**). Thus, kidney failure in *xor* KO mice may underlie our observed plasma accumulation of tryptophan oxidation products and urea cycle metabolites and related species. Finally, serum levels of the TCA cycle intermediates citrate, fumarate, oxaloacetate and malate have been reported to be significantly increased in human renal disease patients and appear to be correlated with disease progression and severity [47]. Thus, the elevated level of TCA cycle intermediates we observe in *xor* KO mice is in accord with that seen in human renal disease patients and may also be a consequence of kidney dysfunction, contributing to the failure of *xor* KO mice to thrive.

Plasma phospholipids, especially phosphatidylcholines and ceramides were observed to increase in *xor* KO mice, but not in allopurinol treated WT mice (**Fig. S1)**. Increased plasma phosphatidylcholines in KO mice is in accord with a prior report demonstrating an essential developmental role for *xor* gene expression during adipogenesis in mouse embryos [33]. Enhanced expression of adipogeneis-related genes was previously described in *xor*-disrupted mice with the accumulation of glyceride-rich lipids in the renal tubules [27]. In contrast, allopurinol treatment was not found to inhibit adipogenesis or lipid-droplet formation in a human XOR transfected pre-adipocyte cell line [33], raising the possibility that the adipogenic activity of *xor* may be limited to a discrete period during embryonic development. Alternatively, the adipogenic activity of *xor* may be mediated by the XDH activity of XOR, which is not inhibited by allopurinol. Notably, mammalian XOR exists in two interconvertible forms, XO and XDH, with the latter being the predominant form *in vivo*. Allopurinol, and its active metabolite oxypurinol, inhibits the XO activity by binding to the molybdenum site of the enzyme [48], but not the FAD site of XDH which has a higher NADH oxidizing activity. Given that XDH activity remains unperturbed with allopurinol treatment, increased phospholipids in *xor* KO suggests the possible mediation by the NADH oxidizing activity of XDH for adipogenesis and PPAR-ã activity.

The broad metabolic consequences we observed with high-dose acute allopurinol treatment in mice warrants consideration for allopurinol use in patients. Initially limited to the treatment of patients with gout, allopurinol therapy has since been extended to chronic stable angina [49], stroke [50], congestive heart failure [51] and even chronic kidney disease [52]. Animal studies have also demonstrated efficacy of allopurinol therapy in type II diabetic mice [53] and in kidney diseases with a significant tubulointerstitial component [54], among others. Escalating doses of allopurinol have been advocated by some investigators, even in the face of recognized renal insufficiency [49], [55], [56]. Although allopurinol intolerance is usually mild, it occurs in 10–15% of patients [57] and only in rare cases has more severe complications been reported. Among those more severe complications are Stevens-Johnson and hypersensitivity syndromes [57], DRESS (drug reaction and eosinophilia syndrome) [58] and an increase in the number of opportunistic infections in patients with inflammatory bowel disease who receive allopurinol in combination with thiopurine therapy [59]. Admittedly, severe adverse effects of allopurinol in patients are rare; however subclinical renal consequences may be more widespread. The data presented herein provide a compendium of plasma biomarkers that may be used to identify subclinical toxicity of allopurinol and also a cautionary note for periodic monitoring of kidney function in allopurinol-treated subjects.

In summary, the present study demonstrates the broad efficacy of untargeted plasma metabolite profiling to render a systems biology perspective on the consequences of XOR enzyme inactivation, resulting from either gene deletion or pharmacological inactivation. This comprehensive perspective goes well beyond providing molecular identities of primary XOR substrates and products, by additionally informing on the activity of pathways that are linked to these molecules and the functional integration of XOR with more distal metabolic pathways and physiological systems. Untargeted metabolite profiling provides an unprecedented tool to illuminate the often obscure *in vivo* push-pull connectivity of metabolic pathways and systemic consequences of pathway perturbations.

## Materials and Methods

### Animals and Reagents

The animal study protocol was in accordance with the National Institutes of Health Guide for the Care and Use of Laboratory Animals and approved by the New York Medical College Institutional Animal Care and Use Committee. XOR+/− mice were obtained from Dr. Toren Finkel (NIH/NHLBI) or purchased from an NIH breeding facility and bred locally. Homozygous XOR knockout mice were obtained by screening progeny born to heterozygous parents, followed by PCR amplification using RedTaq ReadyMix PCR reaction mix (Sigma, Saint Louis, MO) as described in *Supplementary Information Legends* (**Text S1**). LC-MS grade acetonitrile (ACN) and ddH_2_O were purchased from Fischer Scientific. OmniTrace glacial acetic acid and formic were obtained from EMD Chemicals. All other chemicals and standards were obtained from Sigma Aldrich in the best available grade.

### Sample Preparation for Metabolite Profiling

Plasma samples were diluted 1∶25 with 70% ACN in ddH_2_O containing 0.2% acetic acid. The diluted samples were briefly vortexed and centrifuged for 5 min at 16,000×g to pellet precipitated proteins. For aqueous normal phase (ANP) chromatography [15], sample supernatants were directly transferred to autosampler vials for analysis by HPLC-MS. For reversed phase (RP) chromatographic separation, supernatants were dried down under vacuum, then resuspended with 5% ACN in ddH_2_O containing 0.1% formic acid before analysis by HPLC/MS.

### LC-MS and LC-MS/MS Platforms for Metabolite Profiling

Untargeted metabolite profiling was performed using both ANP and RP chromatographic separations (for resolution of polar and nonpolar compounds, respectively), dual spray electrospray ionization and high resolution accurate mass determination using a time-of-flight (TOF) mass spectrometer. Notably, LC-MS produces a continuous multidimensional data matrix, consisting of retention time, mass to charge ratio, and ion abundance.

The LC system comprised a Cogent Diamond Hydride™ (ANP) column (2.1×150 mm, 3.5 µm particle size; Microsolv Technology Corp, Eatontown, NJ), a Zorbax SB-AQ (RP) column (2.1×100 mm, 1.8 µm particle size, Agilent Technologies, Santa Clara, CA), and a Model 1200 Rapid Resolution LC system consisting of a binary pump, on-line degasser, thermostated dual 54-well plate autosampler and a thermostated column compartment (Agilent Technologies, Santa Clara, CA). A precolumn replacement filter frit (0.5 µm, Upchurch Scientific, Oak Harbor, WA) and rapid resolution cartridge (Eclipse XDB-C8, Agilent technologies) were placed in front of the ANP and RP columns, respectively, to prevent column clogging. The LC flow was coupled to an Agilent model 6230 accurate mass time-of-flight (TOF) mass spectrometer, equipped with dual spray electrospray ionization (ESI) source. A separate isocratic pump was used deliver an internal reference mass solution (ions m/z 121.0509 and 922.0093) to the second ESI source for continuous mass calibration during sample analysis. An Agilent 6538 UHD Accurate Mass Q-TOF with same ANP and RP platform was used to conduct fragmentation analysis for confident molecular identification. LC parameters were set as follows for ANP separation: 5 µl injection volume, 0.4 ml/min mobile phase flow rate, 25°C column temperature and 5°C autosampler temperature. The mobile phase consisted of 0.2% acetic acid in ddH_2_O (solvent A) and 0.2% acetic acid in ACN (solvent B). Gradient steps were applied as follows: 0–2 min, 85% B; 2–3 min, to 80% B; 3–5 min, 80% B; 5–6 min, to 75% B; 6–7 min, 75% B; 7–8 min, to 70% B; 8–9 min, 70% B; 9–10 min, to 50% B; 10–11 min, 50% B, 11.0–11.1 min, 20% B; 11.1–14 min, 20% B; 14–14.1 min, 5% B; 14.1–24 min, 5% B; 24–24.1 min, 85% B and 24–34 min, 85% B. Both positive and negative mass spectra were acquired in 2 GHz (extended dynamic range) mode with 1.41 spectra/sec sampled over a mass/charge range of 50–1000 Daltons. The TOF capillary voltage was set at 4000 V for positive ions and 3500 V for negative ions with the fragmentor set to 175 V. The nebulizer pressure was 35 psi and the nitrogen drying gas was 250°C, delivered at a flow rate of 12 l/min. Data was saved in centroid mode using Agilent MassHunter Workstation Data acquisition Software (revision B346). For RP separation, the mobile phase consisted of 0.1% formic acid in H2O (solvent A) or ACN (solvent B). The gradient was as follows: 0–2 min, 5% B; 2–17 min, 98% B; 17.1–27 min, 98% B; 27.1–37 min, 5% B. Other LC and TOF parameters used for RP chromatography were the same as for ANP. To minimize potential salt and other contaminants in the ESI source, a time segment was set for both ANP and RP positive and negative acquisitions that directed the first 0.2 ml of column elute to waste.

### Data Processing and Analysis

Raw data files were processed using Agilent MassHunter Qualitative Analysis Software (version B346). Untargeted molecular feature extraction (MFE) [16], [60] generates features (compounds) based on the elution profile of identical mass and retention times, within a defined mass accuracy (<5 ppm). These features are further grouped into one or more “compounds” based on their isotope pattern, the formation of dimer, adduct ions (e.g. H^+^, Na^+^, K^+^ for positive mode and H^−^, CH_3_COO^−^, HCOO^−^ and Cl^−^ for negative ion mode) and common neutral losses of H_2_O and NH_3_. Compounds/features with absolute peak heights of 1000 or greater were selected and stored as compound exchange format (CEF) files for feature alignment, data processing and multivariate statistical analysis in Mass Profiler Professional (Agilent Technology, MPP, version B2.02). Each aligned mass was associated with its neutral mass, ion intensity and retention time. The aligned data were filtered, considering only features that were detected in all biological replicates from at least one sample group. To complement the untargeted MFE findings and reduce false-positive and false-negative detection rates, we further conducted recursive analysis, wherein a composite list of ions found by MFE were targeted for re-extraction against raw data for all potential ion species (isotopes, adducts, dimers and trimers). The recursive CEF files were re-imported into MPP to provide a higher confidence analysis. Notably, recursive analysis is effective for finding missing features that would otherwise result in overlooking actual resident metabolites by subsequent statistical analysis.

### Statistical Analysis

MPP was used to provide a multivariate statistical platform for comparative metabolite profiling. Principal component analysis (PCA) and hierarchical cluster analysis (HCA) are two unsupervised pattern recognition algorithms used to examine data sets for expected and unexpected clusters, including the presence of outliers without prior sample grouping information. Aligned features detected in all biological replicates of at least one group were directly applied for a 3-dimentional visualization of the data. One-way analysis of variance (ANOVA) was applied to find metabolites showing statistical differences across groups. The Benjamini Horchberg correction was applied to adjust for false-positive discovery, arising from multiple testing of p-values (adjusted for predicted p<0.05).

### Differentially-expressed Metabolite Identification

A critical step in metabolite profiling is identification of unknown metabolites. Differential metabolites with fold changes greater than 2, compared to WT, were initially searched against an in-lab annotated METLIN Personal Metabolite Database (Agilent Technologies), based on accurate monoisotopic neutral masses (<5 ppm). A molecular formula generator (MFG) algorithm in MPP was used to generate and score empirical molecular formulae based on a weighted consideration of monoisotopic mass accuracy, isotope abundance ratios, and spacing between isotope peaks. Notably, MFG imposes additional constraints on the list of candidate molecular formulas detected by a METLIN database search. A putative compound ID was tentatively assigned when METLIN and MFG concurred for a given candidate. Tentatively assigned compounds were verified based on a match of LC retention time and/or MS/MS fragmentation patterns to pure molecular standards. Fragmentation pattern matches were performed for the identification of positional isomers that were unresolved by HPLC and for relatively high molecular weight compounds (600–1,000 Da), where “hits” based on MFG alone can exceed 100 and thus preclude confident molecular identification if considered alone.

### Kidney Histology and Assays

Kidneys (from 3–5 mice for each *xor* genotype) were fixed in 4% paraformaldehyde and embedded in paraffin. Sections (3–4 microns) were stained with hematoxylin/eosin (H&E), periodic acid-Schiff reagents (PAS) and Masson’s trichrome for routine histology. Xanthine and hypoxanthine crystals were visualized by darkfield and polarized light microscopy.

## Supporting Information

Figure S1 Box-whisker plot depicting metabolites that change significantly in *xor* KO vs. WT mice (P<0.05), but not significantly different in WTA (allopurinol-treated) vs. WT mice. The bottom and top of the box denote the 25^th^ and 75^th^ percentile of the ion intensity. The whiskers represent the maximum and minimum of the data. The median and mean are represented as solid and dashed lines, respectively, within each box.
(TIFF)

Figure S2 Curated network, depicting the global metabolic consequences of XOR gene deletion. Pathways are plotted using PathVisio (http://pathvisio.org/), a non-proprietary online access tool for displaying and editing biological pathways. Metabolic linkages are from KEGG pathway maps (http://www.genome.jp/kegg-bin/show_organism?menu_type=pathway_maps&org=mmu and presented using the following nomenclature to denote biological entities: gene products, black font in a black box; metabolites, blue font in a blue box. The pathway is further annotated with KEGG IDs of metabolites and the Entrez gene IDs of gene products. Observed XOR-knockout associated changes in metabolite expression are quantified as Log_2_ fold-change, relative to XOR wildtype control, and denoted in green for molecules with levels that are upregulated and red for molecules that are downregulated. Metabolites without annotated fold-changes were either undetected by LC-MS or exhibited no significant change from control levels.
(TIFF)

Text S1 PCR screening of murine xor genotypes.
(DOCX)
