# Supplementary figures and images for: Untargeted Plasma Metabolite Profiling Reveals the Broad Systemic Consequences of Xanthine Oxidoreductase Inactivation in Mice
Source: PLoS One. 2012 Jun 18;7(6):e37149. doi: 10.1371/journal.pone.0037149 (PMC3377762; doi:10.1371/journal.pone.0037149)

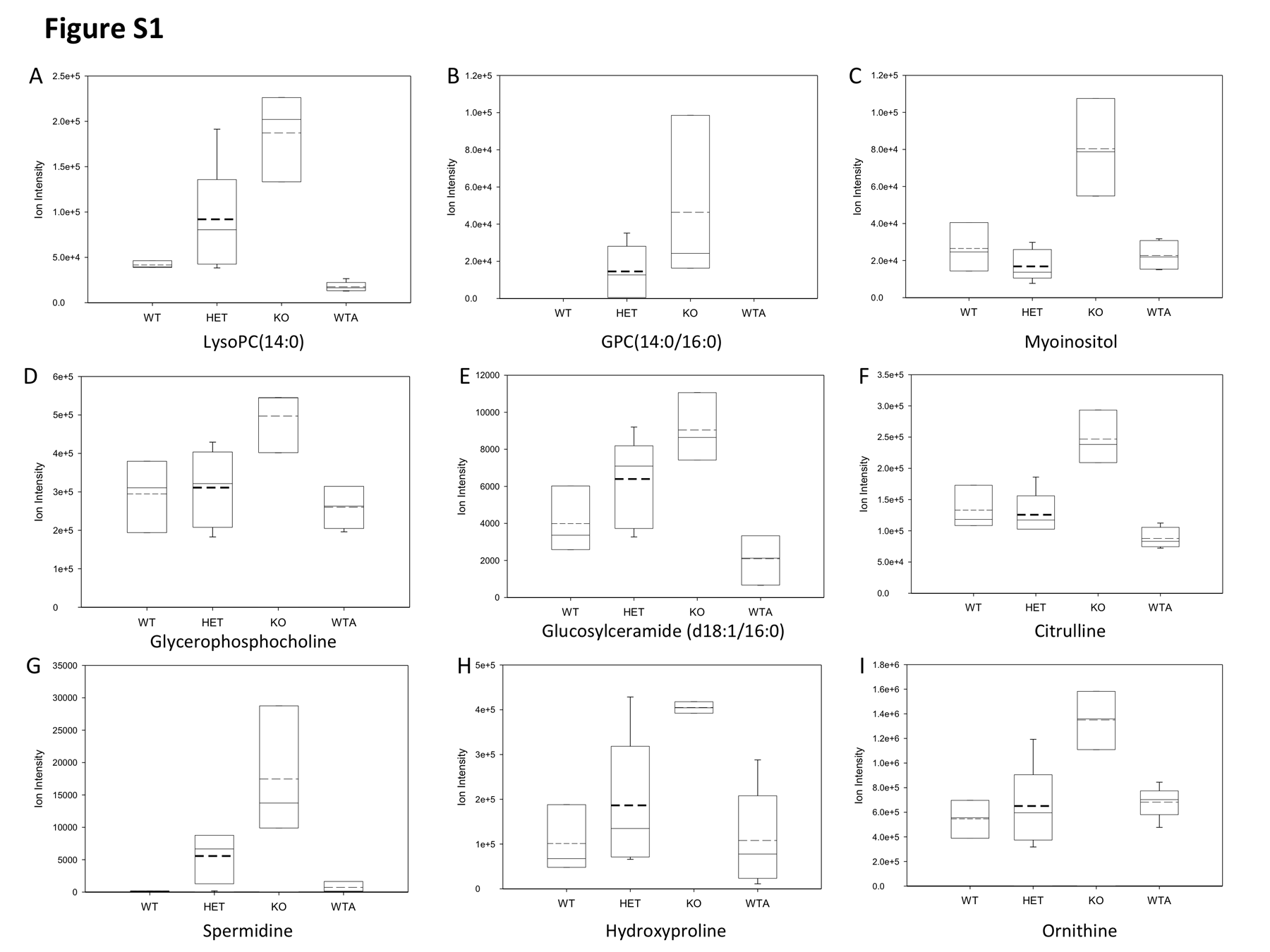

Supplement: Figure S1 — Box-whisker plot depicting metabolites that change significantly in xor KO vs. WT mice (P<0.05), but not significantly different in WTA (allopurinol-treated) vs. WT mice. The bottom and top of the box denote the 25th and 75th percentile of the ion intensity. The whiskers represent the maximum and minimum of the data. The median and mean are represented as solid and dashed lines, respectively, within each box. (TIFF) [file pone.0037149.s001.tif]

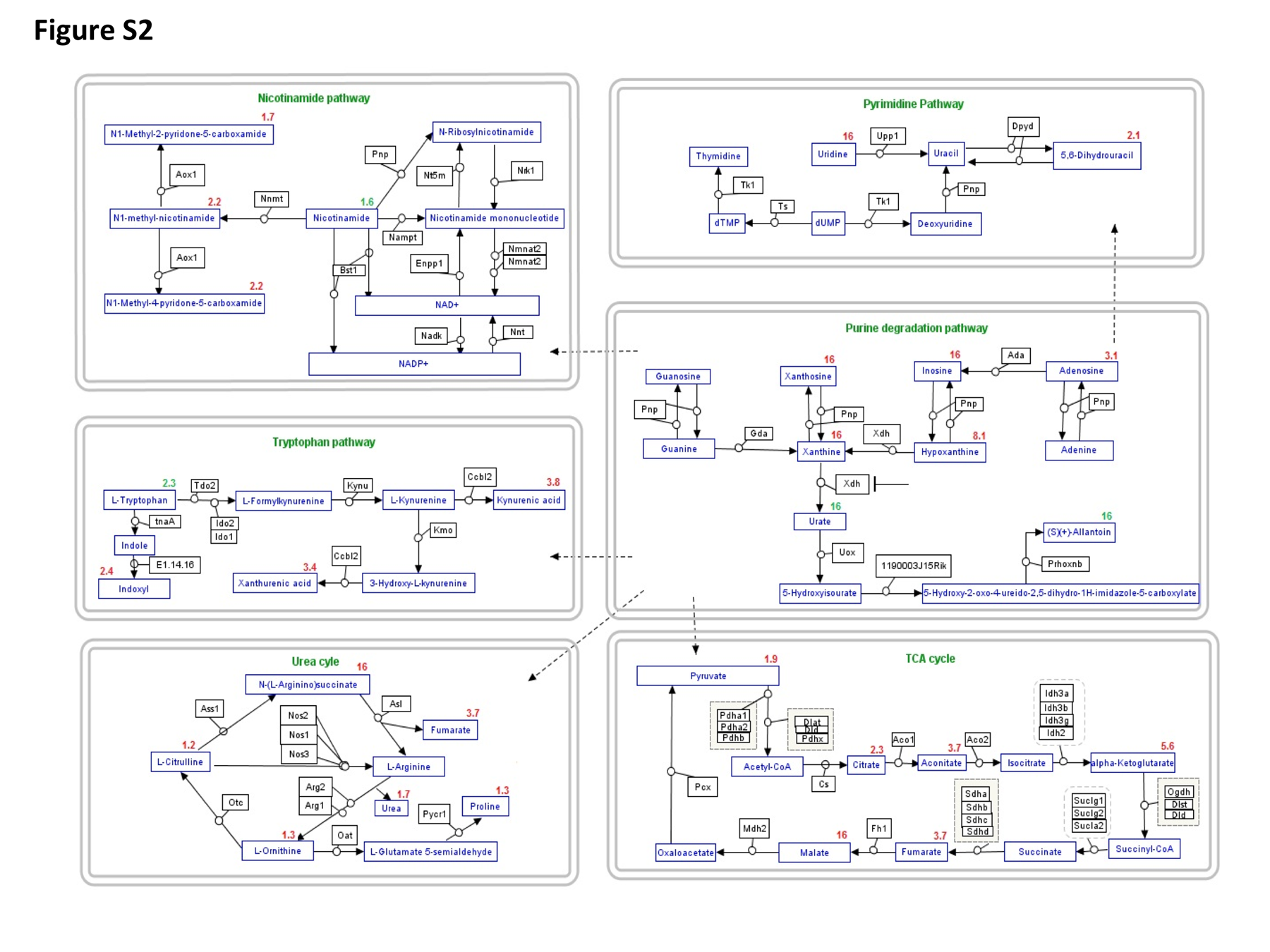

Supplement: Figure S2 — Curated network, depicting the global metabolic consequences of XOR gene deletion. Pathways are plotted using PathVisio (http://pathvisio.org/), a non-proprietary online access tool for displaying and editing biological pathways. Metabolic linkages are from KEGG pathway maps (http://www.genome.jp/kegg-bin/show_organism?menu_type=pathway_maps&org=mmu and presented using the following nomenclature to denote biological entities: gene products, black font in a black box; metabolites, blue font in a blue box. The pathway is further annotated with KEGG IDs of metabolites and the Entrez gene IDs of gene products. Observed XOR-knockout associated changes in metabolite expression are quantified as Log2 fold-change, relative to XOR wildtype control, and denoted in green for molecules with levels that are upregulated and red for molecules that are downregulated. Metabolites without annotated fold-changes were either undetected by LC-MS or exhibited no significant change from control levels. (TIFF) [file pone.0037149.s002.tif]
