# Supplementary material for: Untargeted Plasma Metabolite Profiling Reveals the Broad Systemic Consequences of Xanthine Oxidoreductase Inactivation in Mice
Source: PLoS One. 2012 Jun 18;7(6):e37149. doi: 10.1371/journal.pone.0037149 (PMC3377762; doi:10.1371/journal.pone.0037149)
Supplement: Text S1 — PCR screening of murine xor genotypes. (DOCX) [file pone.0037149.s003.docx]

Transgenic mice were identified at the age of 2 weeks using PCR primers specific for the amplification of *xor* wild-type and knockout transgenes from genomic DNA that was purified from mouse tail tissue using a DNA purification kit (Puregene, Minneapolis, MN). PCR employed primers as follows: Primer A (knockout), 3'- CTA TTC GGC TAT GAC TGG GC -5'; primer B (knockout), 5'-ATG CGA TGT TCG CTT GGT GG-3'; primer C (wild type), 5'-CCT ATG CCT TCC ACA GTT GT -3' primer D (wild type), 3'- CAC CGT GAT GAT CTC CAA GT-5'. Primers A and B are specific for *xor* knockout gene amplification, yielding a 350-bp fragment, whereas primers C and D are specifically amplify the wild-type mouse *xor* locus to yield a 1283-bp fragment. The PCR conditions were: 94°C for 3 min followed by 35 cycles at 94°C for 30 s, 60°C for 30 s, and 72°C for 3 min. Cycling was followed by a final extension step at 72°C for 7 min. Amplification products were analyzed using 1% agarose gels. For selective pharmacological inhibition of XOR enzyme activity, mice were treated with allopurinol at a daily oral dose of 10 mg/kg for 7 days prior to plasma collection. For metabolite profiling, mouse plasma was acquired by cardiopuncture at ≈2 weeks of age. Two sets of murine plasmas were analyzed in this study by untargeted metabolite profiling. The first set compared 5 *xor* KO mice and 5 *xor* wildtype (WT) mice. A second independent study compared the plasma metabolomes of 4 mouse groups: *xor* WT, *xor* HET, *xor* KO and *xor* WT mice following allopurinol treatment (WTA). All mice were kept in a temperature-controlled room at 23°C under a 12h light/dark cycle with food and water available *ad* *libitum*.
